# Prevention of Congenital Cytomegalovirus Infection with Vaccines: State of the Art

**DOI:** 10.3390/vaccines9050523

**Published:** 2021-05-19

**Authors:** Susanna Esposito, Giulia Chiopris, Giulia Messina, Tiziana D’Alvano, Serafina Perrone, Nicola Principi

**Affiliations:** 1 Pediatric Clinic, Pietro Barilla Children’s Hospital, University of Parma, Via Gramsci 14, 43126 Parma, Italy; giulia.chiopris@gmail.com (G.C.); messina.giulia@gmail.com (G.M.); tiziana.dalvano@gmail.com (T.D.); 2 Department of Medicine and Surgery, Neonatology Unit, Pietro Barilla Children’s Hospital, University of Parma, 43126 Parma, Italy; Serafina.perrone@unipr.it; 3 University degli Studi di Milano, 20122 Milan, Italy; nicola.principi@unimi.it

**Keywords:** CMV, CMV infection, congenital infection, vaccination, vaccine

## Abstract

Cytomegalovirus (CMV) is the most common cause of congenital infection and non-genetic sensorineural hearing loss in childhood. Up to 2% of neonates, with the highest percentages found in developing countries, are congenitally infected with CMV. At birth, most of these infants are asymptomatic. However, approximately 10% have signs and symptoms of the disease, and 40–60% of symptomatic neonates will later develop permanent neurologic sequelae. To reduce congenital CMV (cCMV) infection, a vaccine able to prevent primary infection is essential. In this narrative review, actual ongoing research about the development of a CMV vaccine is discussed. The progressive increase in knowledge on the ways in which the host’s immune system and CMV relate has made it possible to clarify that the development of a vaccine that is certainly capable of reducing the risk of cCMV infection, and preventing both primary and nonprimary infections is extremely difficult. Many of the ways in which the virus evades the immune system and causes cCMV infection are not yet fully understood, especially in cases of nonprimary infection. Moreover, the schedule that should be recommended and that subjects must be vaccinated to obtain the greatest effect have not been precisely defined. Further studies are needed before the problem of cCMV infection and its related challenges can be totally solved.

## 1. Background

Cytomegalovirus (CMV) is the most common cause of congenital infection and non-genetic sensorineural hearing loss in childhood (SNHL) [1]. Up to 2% of neonates, with the highest percentages found in developing countries, are congenitally infected with CMV. At birth, most of these infants are asymptomatic. However, approximately 10% have signs and symptoms of the disease. Such infants can be small for gestational age and present with hepatosplenomegaly, jaundice, petechiae, chorioretinitis, and microcephaly [1]. Moreover, 40–60% of symptomatic neonates will later develop permanent neurologic sequelae, including cerebral palsy, cognitive impairment, microcephaly, and SNHL [2]. Those who are born asymptomatic are also at risk of developing sequelae, as 6–23% of them will eventually develop SNHL, and up to 5% will have neurological problems [3]. Placental transmission of CMV can occur after a primary maternal infection, i.e., when seronegative mothers contract a CMV infection during pregnancy or after a nonprimary infection [4,5,6]. In this case, congenital CMV (cCMV) infection occurs in children from already-seropositive mothers as a result of either reactivation of the virus or reinfection with a new CMV strain. A great number of women of childbearing age are already seropositive for CMV. This explains why most cCMV infections depend on a nonprimary infection. However, it has repeatedly been reported that the risks of cCMV infection and severe cCMV disease are significantly higher after primary infection than after nonprimary infection. The risk of viral placental transmission was found to be higher than 70% and between 0.4% and 6% in primary and nonprimary infections, respectively. Moreover, it has been reported that after a mean follow-up of 4.7 years, children born to mothers with primary infections had one or more sequelae in 25% percent of the cases, compared to 8% of children born to women with preconceptual CMV immunity [7,8,9,10]. This indicates that pre-existing immunity, although not adequate to exclude cCMV infection, plays a relevant role in reducing the risk of transplacental CMV passage and foetal infection. Moreover, the findings highlight that to reduce cCMV infection, a vaccine able to prevent primary infection is essential, but to obtain the best results, the vaccine should be able to significantly interfere with the mechanisms that permit the development of nonprimary infection [10].

For many years, poor attention has been given to the development of CMV vaccines. It is only since 2000, when the National Academy of Medicine of the USA stated that the development of a CMV vaccine should have been considered a priority [11], that several vaccines have been developed [12,13]. The evidence that CMV infection is the most common infectious complication of both solid organ and haematogenous stem cell transplantation, and that antiviral prophylaxis and/or treatment have only partial success in transplanted patients developing CMV disease [14,15], has further boosted the research for an effective CMV vaccine. 

In this narrative review, actual ongoing research about the development of a CMV vaccine is discussed. Systematic searches were performed on PubMed, Google Scholar, clinicaltrials.gov, and pharmaceutical company websites. We considered papers with “CMV infection”, “CMV vaccines”, “congenital CMV infection”, “vaccination against CMV”, “infants”, “transplant patients”, and “public health” as keywords, published up to February 2021. Language was restricted to English.

## 2. Immune Response of the Host to CMV Infection 

To understand how a CMV vaccine should be formulated with scientific bases for assuring potential clinical efficacy for cCMV infection prevention, the characteristics of the immune response against CMV must be taken into account. Although the immune mechanisms that engage CMV at the maternal–foetal interface remain yet to be fully understood, studies have shown that both innate and adaptive immune responses are activated and are effective in the case of a primary CMV infection, and that to control reactivation and the development of severe CMV disease, long-lasting immunity is required [12]. After primary infection, natural killer (NK) cells are immediately activated, inflammatory cytokines are released, and infected cells are lysed. Multiple NK-mediated antiviral mechanisms have been demonstrated, including the increased expression of ICAM-1 and LFA-3 by infected cells, which renders them more susceptible to NK killing [16]. The importance of NK cells in controlling CMV infection is strongly supported by evidence that in subjects without NK cells, severe CMV disease can develop [17]. However, CMV can evade NK cell killing through mechanisms mediated by the products of the UL16, UL18, UL40, and UL141 viral genes that suppress the presentation of ligands for the NK cell activating receptor NKG2D on the cell surface [18]. As the mechanisms that confer CMV resistance to NK cell recognition may be lost during in vitro prolonged passage of the virus in cultured cells, this can have relevance for live attenuated vaccine design [19]. On the other hand, vaccines capable of modulating or abolishing the expression of genes that limit NK cell activity on CMV could be relevant in reducing CMV infection and cCMV disease [20].

Active viral replication is strongly suppressed by adaptive immunity, which is primarily mediated by CD4 and CD8 T cells. The T cell response is mainly directed against conserved CMV proteins, including the 65 kDa phosphoprotein (pp65) and the immediate early 1 (IE1) protein and has a relevant role in the protection of transplant patients and the prevention of cCMV infection. Recent reports provide evidence that pp65 is involved in downmodulation of the cellular antiviral cytokine response, can cause reduced expression of major histocompatibility complex class II molecules, and can directly interact with NKp30, the NK cell-activating receptor [21]. Practically, pp65 destabilizes the human host response. The IE1 protein counteracts intrinsic and innate host responses that terminate the viral life cycle. In particular, IE1 antagonizes apoptosis, ND10-related transcription silencing, and type I IFN signalling [22]. In both kidney transplant recipients and heart transplant recipients with CMV infection, a valid CMV-specific T cell response was associated in 90% of cases with a low viral load and early infection resolution. In contrast, the opposite was found in patients with a poor T cell response [23]. Similarly, in pregnant women with a primary CMV infection, the cultured ELISPOT response to pp65 was significantly higher in non-transmitting mothers than in those who transmitted the infection to the foetus [24].

Despite efficient cell-mediated immunity, CMV is not cleared, which explains why this infectious agent can enter a latent state and permanently remain in the host in most infected patients. CMV maintains a latent reservoir within bone marrow-derived progenitors of blood monocytes [25]. Several CMV genes (US2, US3, US6, and US11) interfere with cell-mediated immune responses or encode immunomodulatory gene products that can play a role in CMV immune evasion and favour latency. While much remains to be understood about the signalling events and coordination of repressive activities to repress viral gene expression for latency, much less is known about how these layers of control are unravelled for reactivation [26]. Presently, it is thought that reactivation is strictly linked with the expression of two major early proteins, IE72 and IE76, and with the presence of LUNA, UL138, US28, and LAcmvIL-10 [27]. The understanding of this complex system of putative viral immune evasion genes might have implications for CMV vaccine strategies. To be definitively effective, future vaccines should boost cellular immunity against the proteins encoded by these genes, thus limiting the risk of reactivation and development of cCMV infections [28].

Regarding the humoral immune response, the CMV infection is followed by a prompt and large production of antibodies against several CMV proteins, including those already cited for their involvement in cell-mediated immunity (IE1 and pp65), some envelope glycoproteins (gB, gM/gN), and gH/gL complexes. All of the latter proteins are essential for viral entry into epithelial and endothelial cells, and for viral replication [29]. The potency of these antibodies in CMV neutralization varies, with those targeting the complexes being significantly more effective than those directed against gB and gH/gL. Whereas antibodies to gB are mainly non-neutralizing, immunization of experimental animals with the pentamer elicits CMV-neutralizing antibody titres that persist to high levels over time, and that are a hundred- to a thousand-fold higher than those found in individuals who have recovered from a primary CMV infection [30]. Sera from animals immunized with pentamers neutralize cell infection and prevent viral dissemination from endothelial cells to leukocytes. Neutralizing monoclonal antibodies from immunized animals show the same potency as human antibodies and target both the same sites and additional sites on the pentamer [30]. However, it is not precisely defined whether humoral immunity alone is enough to protect the foetus from cCMV infection. Immunoglobulins can prevent foetal CMV infection in rhesus monkeys [31]. It seems likely that this may occur in humans with primary infections, but to prevent cases due to nonprimary infection, a more complex defensive mechanism involving cell-mediated immunity is probably needed.

## 3. CMV Vaccines

The history of CMV vaccines started in the 1970s, when preparations based on live attenuated viruses were developed [32]. It was thought that this was the simplest and most effective method for preparing a vaccine against CMV because live viruses express a full or nearly full complement of viral antigens and can stimulate humoral and cellular immune responses, closely mimicking those induced by natural infection. Experimental findings and human studies greatly dampened the initial expectations, mainly because immune responses were lower than hoped, and the incidence and severity of infection were only partially reduced [32]. Only one of these vaccines was extensively studied, namely the one containing the Towne strain. It was found to be safe and well tolerated. However, when administered to kidney transplant recipients, this vaccine was effective in reducing the clinical manifestation of CMV disease and the risk of graft rejection [32], but was not able to reduce the risk of infection. Similarly, the vaccine failed to prevent CMV infection in healthy women exposed to infected children in day care [33]. Finally, vaccine administration was associated with protection from infection in healthy volunteers exposed to low doses of unattenuated CMV but was less effective than previously acquired natural immunity when higher doses were used [33]. This led to the conclusion that the Towne virus was over-attenuated and probably incapable of conferring sufficient immune responses as a vaccine [34]. To improve its immunogenicity, a recombination of the Towne strain with the non-attenuated Toledo strain was performed, substituting some regions from the wild virus genome with the corresponding regions of the Towne virus genome [35]. Four Towne/Toledo chimaera vaccines were prepared and tested in seronegative men receiving different subcutaneous doses of the new viruses. All the studied preparations were found to be safe and well tolerated. However, despite differences among preparations, recombinant CMV strains were unsuccessful compared to the wild type in enhancing vaccine-elicited immune responses [36]. 

Presently, new attempts to develop a live attenuated CMV vaccine have been made (Table 1). To improve immunogenicity and efficacy, the CMV virus was modified to restore the expression of genes that have been found to be of relevance in the development of protective immunity [36]. A preparation containing a replication-defective CMV virus with restored expression of the gH/gL/pUL128–131 pentameric complex was evaluated for phase I vaccine safety and immunogenicity in CMV-seronegative and CMV-seropositive adults. In seronegative subjects, a significant increase in neutralizing antibody concentrations was evidenced with levels that, one month after the third dose, were comparable with those detected after the natural infection for both neutralizing antibody levels and the cellular response [36]. In particular, antibodies capable of neutralizing epithelial cell infection were detected. As this is a fundamental step for the prevention of human placental cytotrophoblasts, the vaccine was considered worthy of further development, particularly because of the interest in it as a preventive measure against cCMV infection [36].

A major step forward in CMV vaccine development was made when CMV-neutralizing proteins evoked by natural infection were used. Initially, the majority of vaccines incorporated glycoprotein B (gB) in association with an adjuvant. The vaccine with MF59, an oil-in-water adjuvant [37], was shown to be safe, immunogenic, and capable of providing 50% protection against primary infection in seronegative postpartum women [38] and 43% protection in seronegative adolescent girls [39]. Moreover, administration in transplant patients limited the periods of viremia and the need for antiviral treatment [40]. However, the long-term efficacy of the vaccines was debated, as the serum antibody levels and protection waned with time, although they could be boosted by the administration of further doses [41]. Better immunogenicity was found when gB was combined with AS01, an adjuvant containing the immunostimulants 3-O-desacyl-4′-monophosphoryl lipid A and QS-21, which promote Toll-like receptor 4 activity [42]. In this case, higher and more persistent antibody titres were detected [43]. Unfortunately, this preparation was not tested in efficacy trials.

Further progress in CMV vaccine development was made when it was evidenced that administration of a pentameric complex of proteins present on the surface of CMV could induce a greater production of neutralizing antibodies than gB [44]. This structure, consisting of glycoprotein H (gH), glycoprotein L (gL), and the products of genes UL128, 130, and 131, was found to generate the most effective immune response against CMV endothelial and epithelial cell entry [44]. Moreover, in seronegative pregnant women exposed to the virus, it was evidenced that the immune response to this pentameric complex offered the greatest activity against transmission of CMV to the foetus [45].

Potential efficacy for the prevention of cCMV infection was shown by some vaccines that utilize viral vectors to obtain an immune response against some viral components. One of these vaccines, a vaccine based on a canarypox virus expressing gB (ALVAC-CMV), was found to be effective when used as a prime for live-attenuated strains [46]. In subjects receiving the vectored gB vaccine and later immunized with the Towne vaccine, neutralizing antibody titres against gB developed sooner, were much higher in number, and persisted longer than in subjects receiving only gB. Similar canarypox vaccines expressing CMV pp65 induced long-lasting cytotoxic T lymphocyte (CTL) responses in all originally seronegative volunteers, with a CTL precursor frequency similar to that of naturally seropositive individuals [46]. The addition of proteins that evoke neutralizing antibodies, and proteins that strongly stimulate cell-mediated immunity, was considered the best solution for limiting foetal infection and CMV disease in transplanted patients. In a phase II study, a bivalent CMV-DNA vaccine encoding both gB and pp65A was found to elicit robust pp65-specific T cell responses and late gB-specific B cell responses. Presently, a phase III trial assessing its efficacy in the prevention of mortality in seropositive transplant recipients is ongoing [47]. A vaccine based on the modified vaccinia Ankara (MVA) that encodes three immunodominant CMV antigens (pp65, IE1-exon4, and IE2-exon5) was found to be able to safely and durably expand high levels of CMV-specific T cells when tested in a phase 1 trial in healthy adults [48]. Moreover, in a phase II randomized clinical trial, the administration of this vaccine led to a relevant reduction in CMV viremia risk in transplant patients. Reactivation of CMV occurred in 9.8% of vaccine recipients compared to 19.6% of patients given a placebo (hazard ratio, 0.46 [95% CI, 0.16 to 1.4]; *p*= 0.075). Levels of long-lasting, pp65-specific T cells with the effector memory phenotype were significantly higher in subjects with the vaccine than in the placebo group [49]. 

Recently, several peptide, DNA, and mRNA vaccines coding for pp65, gB, or other relevant CMV proteins have been studied to improve immunogenicity and efficacy [50,51,52,53,54,55,56]. Some of them have shown preliminary evidence of efficacy in transplant recipients. A CMV gB nucleoside-modified mRNA vaccine elicited an antibody response with greater durability and breadth than in the MF59-adjuvanted gB protein immunization [57]. However, the most advanced mRNA preparation is the mRNA-1647 vaccine. It comprises five mRNAs encoding the subunits of the pentamer complex and one mRNA encoding the gB target antigen. Phase I (NCT03382405) and phase II (NCT04232280) studies specifically devoted to evaluating the immunogenicity, safety and tolerability, and the most effective dosage in humans have been carried out and partly completed. Interim analysis of phase II trial results revealed the substantial ability of the vaccine to induce an immune response significantly greater than that evoked by the natural infection of both seronegative and seropositive individuals. This led to the planning of a phase III pivotal study to evaluate vaccine efficacy in the prevention of CMV infection in seronegative women aged 16–40 years [58].

In addition, virus-like particles and nanoparticles can be used for multivalent antigen presentation. A virus-like particle with gB on the surface has shown a surprisingly high induction of neutralizing antibodies in animals. Moreover, pp65-derived peptides combined with a tetanus toxin epitope have exhibited immunogenicity in humans [59].

## 4. Administration of the CMV Vaccine

Although several approaches to the development of an effective CMV vaccine have been followed and, at least in some cases, preparations have been found to be very attractive because of their potential effectiveness, we are far from achieving an ideal vaccine for the prevention of cCMV infection. Presently, it has been shown that some vaccines can prevent the acquisition of CMV by seronegative women, but definitive demonstration that a vaccine can prevent the development of cCMV infection in children born to seronegative or seropositive mothers is lacking. Moreover, the collection of data in this regard is difficult. It has been suggested that the problem could be solved by immunizing women of childbearing age, and then following up with their children at birth, to understand whether they have acquired CMV during pregnancy through the evaluation of CMV excretion in urine and saliva with molecular methods [60]. Even if this protocol could lead to the collection of reliable data, it seems difficult to implement because it is expensive and requires the enrolment of a great number of women who should be followed over the long term while awaiting pregnancy. Moreover, women could be frequently exposed to CMV excreted by young sons [61]. This could create problems in the evaluation of study results, as the size and extent of the exposure could significantly differ among enrolled subjects. Moreover, it cannot be forgotten that the results of studies regarding the immune response to CMV seem to indicate that for total effectiveness against cCMV, two different vaccines may be necessary. 

Prevention of cCMV infection in seronegative women could be obtained using a vaccine able to induce a significant humoral and cellular immune response against the viral components that are highly immunogenic and have relevant effects in CMV disease determination. Vaccines effective against gB, the pentameric protein complex, and pp65 may be able to significantly reduce cCMV infection [62]. In contrast, it seems highly likely that interventions on the mechanisms that are the basis for nonprimary infection are needed to prevent cCMV infection in seropositive pregnant women. However, even when effective vaccines were available, not all the problems related to cCMV infection prevention would be solved. The duration of protection offered by the different CMV vaccines is not yet defined. Long-lasting protection could allow us to suggest the immunization of all preadolescent girls, as is recommended for the human papillomavirus vaccine [62]. In contrast, the vaccines could be suggested for pregnant women or repeatedly administered during the childbearing age range according to the duration of effective immunity. Universal immunization could be planned in the various countries (such as several European nations and the USA) in which the incidence of CMV seropositivity in younger women is relatively low [63]. To reduce the number of vaccine doses used and the related economic and organizational problems, the selection of seronegative women before vaccine administration could be the most opportune strategy in countries with a high incidence of CMV seroconversion in the first years of life. Whether only women of childbearing age must be the target of vaccination or if other individuals must also be immunized has not been established. The risk for CMV infection is increased if women have a role in the direct care of young children [64]. Moreover, there are sexually transmitted infections [65]. This means that one strategy to reduce the risk of cCMV infection is to extend vaccination to younger children and to men. The efficacy of CMV vaccines against CMV strains different from those used for vaccine formulation is not established and must be defined in order to decide whether new vaccine doses have to be administered in case a new strain begins to circulate in a particular country [66]. 

Finally, the real economic importance of vaccination must be calculated. Unfortunately, this factor is unknown. Investigations in this regard have been conducted, but no definitive conclusions can be drawn, as in most cases the studies have several relevant methodological problems. More than 30 years ago, Porath et al. reported that routine immunization could be cost-saving regardless of the vaccine cost [67]. However, the results are highly debatable as they are derived from poorly documented assumptions. The severity of the long-term medical effects of cCMV was considered significantly greater than that generally reported in clinical studies. Consequently, total health care expenses were greater than what was truly needed. Moreover, costs for global cCMV long-term health care were calculated considering the claims submitted to insurance companies and not those actually paid. More recently, Dempsey et al. [68] reported that the immunization of all adolescent females could have had significant clinical and economic advantages, as it could have reduced the total number of children with neurological problems and resulted in savings of $32.3 million for every 100,000 vaccinated women. Unfortunately, these findings can be criticized, as they were calculated considering the debatable prevalence of both total cCMV infections and symptomatic cases. Moreover, the efficacy of a given CMV vaccine was critical. Reported advantages were evidenced only for a vaccine efficacy of 95%. When the efficacy was reduced to less than 61%, no clinical difference between vaccinated and unvaccinated women was shown, and expenses in the vaccinated group were significantly greater. The most recent cost/efficacy study, carried out in France, where CMV seroprevalence in young people is relatively low, has shown that, from the national health insurance perspective, the general immunization of all adolescent women could be more effective than no vaccination or vaccination of only seronegative subjects [69]. However, when the analysis was performed considering higher seroprevalence values, it was shown that with seroprevalence levels >62%, the immunization of only seronegative adolescent women could be more advantageous. As in some other countries, in France the seroprevalence can be higher than that actually reported [70]. This finding highlights how difficult it can be to evaluate CMV vaccine cost/efficacy and strongly indicates the need for further studies in this regard before the vaccine can be recommended.

## 5. Conclusions

More than twenty years ago, the availability of an effective CMV vaccine capable of reducing the risk of cCMV infection, which is a very severe condition, was considered a priority. The progressive increase in knowledge on the ways in which the host’s immune system and CMV relate has made it possible to clarify that the development of a vaccine that is certainly capable of reducing the risk of cCMV infection and preventing both primary and nonprimary infections is extremely difficult. Many of the ways in which the virus evades the immune system and causes cCMV infection are not yet fully understood, especially in cases of nonprimary infection. Moreover, the schedule that should be recommended and for which subjects must be vaccinated to obtain the greatest effect have not been precisely defined. Further studies are needed before the problem of cCMV infection and its related challenges can be totally solved.

## Figures and Tables

**Table 1:** Main CMV vaccines under clinical development.

Vaccine Category	Vaccines	Antigen Used	Adjuvant	Manufacturer	CT Identifier	Phase
Attenuated and DISC vaccines	V160-001	gB, pp65, IE1	Aluminum phosphate or none	MSD	NCT01986010	1
	Towne-			CMV Research	NCT01195571	1
	Toledo			Foundation.		
	Chimera			International		
	Vaccine			AIDS Vaccine	NCT00370006	1
				Institute	NCT00373412	1
	VCL-CT02			UC-SF, Vical		
	Plu Towne					
	CMV					
Recombinant/Subunit vaccines	GSK1492903A	gB	Proprietary	GSK	NCT00435396	1
					NCT01357915	1
	gB subunit	gB	MF59	University	NCT00299260	2
				College,	NCT01883206	2
	gB/MF59	gB	MF59	London	NCT00133497	2
				NIAID	NCT00815165	2
	gB/MF59	gB	MF59	Sanofi Pasteur	NCT00125502	2
Vectored vaccines	AVX601	gB, pp65, IE1	None	AlphaVax, Inc	NCT00439803	1
	HCMV-MVA Triplex		None	(Novartis, GSK)	NCT01941056	1
					NCT02506933	2
		pp65, IE1- exon4, IE2- exon5 gB, pp65 pp65	None None	City of Hope,		
	HB-101			National	NCT02798692	
	ALVAC-pp65			Cancer Institute	NCT00353977	
				Hookipa		
				Biotech		
				NHLBI		
DNA, mRNA vaccines	ASP0113	gB, pp65	CRL 1005BAK	Astellas, Vical	NCT02103426	1
					NCT01903928	2
					NCT01974206	2
					NCT01877655	3
	VCL-CB01	gB, pp65		Astellas, Vical	NCT00285259	2
Peptide Vaccines	CMVpp65- A*0201 peptide; containing either helper T lymphocyte (HTL)	gB,pentamer complex; pp65; T cell epitope fused to either PADRE or CMV tetanus epitope	CRL	Moderna	NCT03382405	1
			1005BAK		NCT04232280	2
			None None None			
	PADRE peptide or tetanus toxoid peptide Tetanus- HCMVpp65 fusion peptide (CMVpp65- A*0201; CMVPepVax)	pp65; T cell epitope fused to tetanus epitope		City of Hope, National Cancer Institute City of Hope, National Cancer Institute	NCT00712634 NCT00722839 NCT01588015 NCT02396134	1 1 1 2

## Data Availability

Not applicable in a review article.
